# The Asian Oceanian Society of Radiology (AOSR) green radiology survey: a catalyst for action

**DOI:** 10.1007/s11604-025-01918-y

**Published:** 2025-12-23

**Authors:** Evelyn Lai Ming Ho, Tetsuya Fukuda, Elaine Yee Ling Kan, Cher Heng Tan, Danny Hing Yan Cho, Chamaree Chuapetcharasopon, Noriyuki Tomiyama

**Affiliations:** 1 Imaging Department, ParkCity Medical Centre, Kuala Lumpur, Malaysia; 2 Department of Radiology, National Cerebral and Cardiovascular Center, Osaka, Japan; 3 Department of Radiology, Hong Kong Children’s Hospital, Kowloon Bay, Hong Kong; 4 Department of Diagnostic Radiology, Tan Tock Seng Hospital, Singapore, Singapore; 5 Lee Kong Chian School of Medicine, Nanyang Technological University, Singapore, Singapore; 6 Department of Diagnostic and Interventional Radiology, Kwong Wah Hospital, Yau Ma Tei, Hong Kong; 7 MedPark Hospital, Bangkok, Thailand; 8 Department of Diagnostic and Interventional Radiology, The University of Osaka Graduate School of Medicine, Osaka, Japan

**Keywords:** Green radiology, Sustainability, Carbon footprint, AOSR, Asia-Oceania

## Abstract

Approximately 5% and 1% of global greenhouse gas emissions originate from the healthcare sector and medical imaging respectively. These are not insignificant. Despite this, the medical field has been slow to adopt sustainable practices. Green radiology is a sustainable, innovative, and responsible approach in radiological practice that focuses on minimising the negative environmental impact of our technologies and procedures. The Asian Oceanian Society of Radiology (AOSR) conducted a survey across Asia-Oceania to better understand current operations and identify opportunities for meaningful progress toward more sustainable radiological practices. From the 123 institutions that responded, it was found that collectively the carbon footprint of Asia-Oceania’s radiological services are high in some of the countries/regions because of the high density of high energy consumption scanners (such as computed tomography and magnetic resonance imaging scanners). Our survey showed that less than half of the 15 societies that responded had specific academic or research activities related to sustainable radiology and less than a third reported that sustainability had been incorporated in the training curriculum. The AOSR is committed to facilitating the sharing of best practices in green radiology and recommends that Asia-Oceania use an “As Low As Reasonably Achievable” principle, traditionally used in radiation safety and extend this in the approach to the issues of environmental impact of radiological services. The AOSR as a cross-regional professional society, has the mandate to partner with our counterpart societies and industry stakeholders, to emphasise the importance of sustainability as a critical agenda, through our various activities. We foresee that influencing radiology leaders in the region to urgently formulate strategies, implement policies towards adopting eco-friendly approaches including reducing inappropriate imaging and encouraging academic efforts will help to reduce the environmental impact in each country/region. A checklist has been included to help kickstart the process.

**Supplementary Information:**

The online version contains supplementary material available at 10.1007/s11604-025-01918-y.

## Introduction

Approximately 5% of global greenhouse gas (GHG) emissions [1] originate from the healthcare sector, making it a significant contributor to climate change. Medical imaging primarily through the manufacture of equipment and the energy needed to power it, contributes about 1% of global GHG emissions [2]. Despite this, the medical field has been slow to adopt sustainable practices. In radiology, the principle of “As Low As Reasonably Achievable” (ALARA) is well established, traditionally guiding efforts to minimize patient radiation exposure during imaging procedures. This approach should be extended to the practice of sustainable radiology to reduce the environmental impact of imaging, image-guided intervention and therapy. Fortunately, green or sustainable radiology is gaining more urgent attention now with the accelerating climate crisis [3–10].

The Asian Oceanian Society of Radiology (AOSR) is primarily a federation of 24 radiological societies. At the time of this survey, the AOSR also had 6 individual members. These are either the diaspora of Asia-Oceania working outside of Asia-Oceania or from Asia-Oceania countries/regions with just a handful, if not just one radiologist. To better understand current operations and identify opportunities for meaningful progress toward greener, more sustainable radiological practices, the AOSR conducted a survey across Asia-Oceania. The findings are intended to catalyse future action.

## Materials and methods

A series of questions in English were drawn up by the AOSR sustainability working group (SWG) and the survey was carried out from October 15, 2024 to April 15, 2025. The survey was converted to Google Form and an email sent out to all the 24 AOSR member society secretariats and also directly to all 6 AOSR individual members at the time of the survey. As the AOSR depended on the society members’ secretariats to distribute the survey, the total number of institutions the survey was sent out to was not known. Each society member secretariat had their preferred way of distribution of the survey, either via email and/or via social applications to their own membership. For privacy and personal data protection reasons, the AOSR does not have direct access to each society members’ membership database.

The SWG had early on made the decision to translate the survey into Japanese with the cooperation of the Japan Radiological Society (JRS) to improve accessibility and for endorsement by the Japan Radiological Society (JRS). As such, the survey in Japanese was then distributed to the 123 training facilities in Japan. Enquiry was also made if translation was needed, to the other society members where English was not widely spoken. However, none of those responded that the survey needed to be translated into their primary vernacular languages.

Participation in the survey was voluntary and their participation implied granting permission for the data generated from the survey to be used for a project or publication. The target respondents were society leaders, Heads/Chairs/Directors/Managers of departments and/or of Residency programs. The survey answers in Japanese were translated back into English for review.

Respondents representing societies (“society-only” responses) answered the survey questions in the section “Education & Academic Activities Under College/Radiological Society”. For those who were responding only for their own institutions (“institution-only” responses), they answered questions in the sections related to their institution (number of high energy consumption equipment, operating days and hours per day for routine bookings); energy and resource consumption as well as audit and research. Those who answered on behalf of their society as well as their institution, responded to the entire survey (“institution-only” and “society-only” responses). The AOSR Green Survey is attached as supplement A.

All responses were anonymized, and the aggregated data did not contain individual level information. The survey answers were analysed using Microsoft Excel. As Asia-Oceania is very diverse, the respondents were classified according to the World Bank Gross National Income (GNI) Per Capita 2023.

**Fig. 1:**
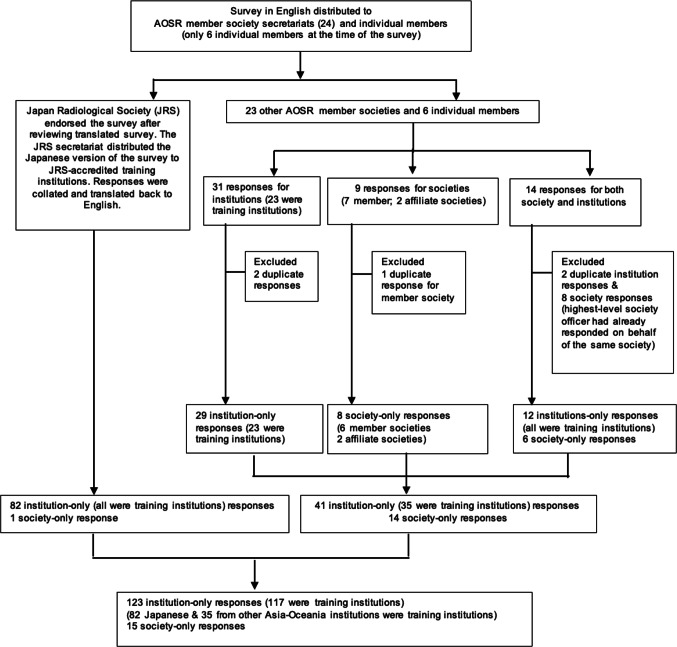
AOSR green survey flow chart

Survey answers were reviewed for completeness and accuracy. Figure 1 shows the inclusion and exclusion criterion. Obvious duplicate responses were excluded. If duplicate responses were noted, yet answers were not identical, the respondent was contacted for clarification, and the correct response recorded. This was possible for society/college related respondents. If more than one survey answer was received from the same institution, the responses from the chair/director/head of department were accepted, the others were excluded for analysis with regard to ‘institution” related questions. If more than one society response was received for those answering on behalf of their institution and society—the highest leadership position such as the President’s/Chair’s responses on behalf of the society was accepted and for the others, only the institution related answers were included for analysis. Responses from affiliate societies of the AOSR member societies were accepted/included as these subspecialty or allied field specialty societies operated autonomously as independent societies. This was also to widen the representation in order to provide a broader assessment of the sustainability landscape in Asia-Oceania.

If there were fields which were not answered, the number of respondents who did not answer the question was noted, if the rest of the respondents’ answers were included for analysis. Nine different Japanese institution respondents each did not answer a question (sub-question) and of the 3 different middle-income country/region (MIC) respondents, 2 did not answer 2 questions, whilst 1 did not answer 1 question.

Institutional review board approval was not needed for this survey as it did not qualify as a human subjects’ research.

## Results

### Institutions

#### Demographics

There were 123 “institution-only” responses of which 6 respondents also completed the survey on behalf of their society (Fig. 1). These “society-only” responses are presented in the society results. Given the relative proportion of the respondents from 13 countries/regions (Table 1), we separated the analysis into Japanese institutions (Japan is a high-income country/region (HIC)), other HIC and middle-income country/region (MIC) institutions. The 4 and 15 respondents from the lower-middle-income and upper-middle income country/region, were then combined into MIC institutions (World Bank GNI Per Capita, 2023).

**Table 1:** Geographic and income level distribution of institution responses

Country/region	All institutions	Only training institutions
High-income country/region (HIC) subtotal	104	100
Australia	2	2
Hong Kong SAR	11	7
Japan	82	82
Oman	1	1
Singapore	7	7
South Korea	1	1
Middle-income country/region (MIC) subtotal	19	17
Upper-middle-income country/region	15	13
Indonesia	1	1
Malaysia	5	4
Thailand	8	8
Tonga	1	0
Lower-middle-income country/region	4	4
Myanmar	1	1
Nepal	1	1
Sri Lanka	2	2
Total number of institutions	123	117

The respondents were from 82 Japanese, 22 other HIC and 19 MIC institutions. The other HIC and MIC institutions were from the rest of Asia-Oceania, with a median of 1.5 respondents per country/region. All (82/82) the Japanese, 89% (18/22) other HIC and 82% (17/19) MIC institutions were training institutions.

The response rate for the survey was 67% (82/123) for the Japanese institutions. The overall response rate for non-Japanese respondents is unknown as the total number of distributed surveys via email and/or social applications by the respective secretariats were not recorded. One out of the 6 AOSR individual members responded (16.7%).

All the Japanese (82/82), other HIC (18/18) and MIC (17/17) training institutions provided both in- and out-patient radiological services. For all the other HIC (4) and MIC (2) non-training institutions, 50% (3/6) provided only out-patient radiological services, i.e. 2 of the HIC and 1 of the MIC.

#### Profile of high energy consumption equipment utilization

In this section, 1 to 2 Japanese institutions did not answer some questions, whilst other HIC and MIC institutions answered all questions. All the Japanese institutions had MRI, CT scanners and IR suites. Two of the other HIC training institutions did not have MRI scanners as well as IR suites and 1 of the MIC training institutions did not have an IR suite. None of the institutions operated 2 or less days per week.

In terms of number of scanners and IR suites, 81% to 96% of Japanese training institutions had 3 or more scanners/IR suites. These institutions operated their scanners 59% (MRI), 68% (CT) and 43% (IR suites) 6 to 7 days a week; 75% (MRI), 70% (CT) and 40% (IR suites) 9 to 16 hours a day (Table 2).

**Table 2:** Profile of high energy consumption equipment utilization: Japanese, other high-income (HIC) and middle-income country/region (MIC) institutions

Institution	Japanese training	Other HIC training	All Other HIC	MIC training	All MIC
Number	82	18	22	17	19
Profile of high energy consumption equipment utilization (MRI)					
Number of scanners					
0 (none)	0/82 (0%)	2/18 (11%)	2/22 (9%)	0 (0%)	1/19 (5%)
1–2	7/82 (9%)	5/18 (28%)	6/22 (27%)	8/17 (47%)	9/19 (47%)
3–4	52/82 (63%)	8/18 (44%)	11/22 (50%)	6/17 (35%)	6/19 (32%)
5–6	19/82 (23%)	2/18 (11%)	2/22 (9%)	1/17 (6%)	1/19 (5%)
>6	4/82 (5%)	1/18 (6%)	1/22 (5%)	2/17 (12%)	2/19 (11%)
Number of days of operation per week					
3–5	34/82 (41%)	5/16 (31%)	5/20 (25%)	3/17 (18%)	4/18 (22%)
6–7	48/82 (59%)	11/16 (69%)	15/20 (75%)	14/17 (82%)	14/18 (78%)
Number of hours of operation per day					
≤ 8	19/80 (24%)	1/16 (6%)	4/20 (20%)	3/17 (18%)	4/18 (22%)
9–16	60/80 (75%)	15/16 (94%)	15/20 (75%)	11/17 (65%)	11/18 (61%)
> 16	1/80 (1%)	0/16 (0%)	1/20 (5%)	3/17 (18%)	3/18 (17%)
Remarks	2 respondents did not answer				
Profile of high energy consumption equipment utilization (CT)					
Number of scanners					
0 (none)	0/80 (0%)	0/18 (0%)	0/22 (0%)	0/17 (0%)	1/19 (5%)
1–2	3/80 (4%)	5/18 (28%)	6/22 (27%)	5/17 (29%)	6/19 (32%)
3–4	45/80 (56%)	9/18 (50%)	12/22 (55%)	8/17 (47%)	8/19 (42%)
5–6	27/80 (34%)	3/18 (17%)	3/22 (14%)	3/17 (18%)	3/19 (16%)
> 6	5/80 (6%)	1/18 (6%)	1/22 (5%)	1/17 (6%)	1/19 (5%)
Remarks	2 respondents did not answer				
Number of days of operation per week					
3–5	26/80 (33%)	1/18 (6%)	1/22 (5%)	3/17 (18%)	3/18 (17%)
6–7	54/80 (68%)	17/18 (94%)	21/22 (95%)	14/17 (82%)	15/18 (83%)
Remarks	2 respondents did not answer				
Number of hours of operation per day					
≤ 8	19/82 (23%)	4/18 (22%)	5/22 (23%)	2/17 (12%)	3/18 (17%)
9–16	57/82 (70%)	13/18 (72%)	15/22 (68%)	10/17 (59%)	10/18 (56%)
> 16	6/82 (7%)	1/18 (6%)	2/22 (9%)	5/17 (29%)	5/18 (28%)
Profile of high energy consumption equipment utilisation (IR)					
Number of suites					
0 (none)	0/82 (0%)	2/18 (11%)	4/22 (18%)	1/17 (6%)	3/19 (16%)
1–2	16/82 (20%)	6/18 (33%)	8/22 (36%)	9/17 (53%)	9/19 (47%)
3–4	37/82 (45%)	9/18 (50%)	9/22 (41%)	4/17 (24%)	4/19 (21%)
5–6	26/82 (32%)	0/18 (0%)	0/22 (0%)	2/17 (12%)	2/19 (11%)
> 6	3/82 (4%)	1/18 (6%)	1/22 (5%)	1/17 (6%)	1/19 (5%)
Number of days of operation per week					
3–5	47/82 (57%)	5/16 (31%)	6/18 (33%)	11/16 (69%)	11/16 (69%)
6–7	35/82 (43%)	11/16 (69%)	12/18 (67%)	5 /16 (31%)	5/16 (31%)
Number of hours of operation per day					
≤ 8	46/81 (57%)	8/16 (50%)	10/18 (56%)	8/16 (50%)	8/16 (50%)
9–16	32/81 (40%)	8/16 (50%)	8/18 (44%)	6/16 (38%)	6/18 (38%)
> 16	3/81 (4%)	0/16 (50%)	0/18 (0%)	2/16 (13%)	2/18 (13%)
Remarks	1 respondent did not answer				

For the other HIC training institutions, 56% to 73% had 3 or more scanners/IR suites. These institutions operated their scanners 69% (MRI), 94% (CT) and 69% (IR suites) 6 to 7 days a week; 94% (MRI), 72% (CT) and 50% (IR suites) 9 to 16 hours a day. For the MIC training institutions, 42% to 71% had 3 or more scanners/IR suites. These institutions operated their scanners 82% (MRI), 82% (CT) and 31% (IR suites) 6 to 7 days a week; 65% (MRI), 59% (CT) and 38% (IR suites) 9 to 16 hours a day. The pattern of number and utilization of scanners were similar whether only training or non-training other HIC and MIC institutions were considered (Table 2).

#### Energy and resource consumption

For Japanese training institutions’ environmentally conscious practices, the figures were above 50% in the responses except for CT scanners with auto-shutdown function (48%) and as high as 99% for paperless radiology requests and reports (Table 3). For the question on policy or practice of recycling of non-contaminated waste in the workplace, the answer does not appear separately for Japanese institutions. The translated term ‘waste management’ in the Japanese context incorporated reuse, recycling and disposal practices for the question on ‘considering environmental factors in making procurement and/or service contract decisions including proper waste management decisions’.

**Table 3:** Energy and resource consumption: Japanese, other high-income (HIC) and middle-income country/region (MIC) institutions

Institution	Japanese Training	Other HIC Training	All Other HIC	MIC Training	All MIC
Number	82	18	22	17	19
Considered environmental factors in making procurement (such as energy efficiency) and/or service contract (proper waste management) decisions					
Yes	44/81 (54%)	14/18 (78%)	18/22 (82%)	10/15 (67%)	12/17 (71%)
No	37/81 (46%)	4/18 (22%)	4/22 (18%)	5/15 (33%)	5/17 (29%)
Remarks	1 respondent did not answer			2 respondents did not answer	2 respondents did not answer
Regularly turned off idle workstations (when not in use)					
Yes	59/81 (73%)	10/18 (56%)	13/22 (59%)	14/16 (88%)	16/18 (89%)
No	21/81 (26%)	7/18 (39%)	8/22 (36%)	13/16 (13%)	2/18 (11%)
**Unsure/*Sometimes	**1/81 (1%)	1/18 (6%)	*1/22 (5%)	0/16 (0%)	0/18 (0%)
Remarks	1 respondent did not answer			1 respondent did not answer	1 respondent did not answer
CT scanners with auto-shutdown function					
Yes	39/81 (48%)	5/18 (28%)	6/22 (27%)	5/16 (31%)	6/17(35%)
No	36/81 (44%)	12/18 (67%)	15/22 (68%)	11/16 (69%)	11/17 (65%)
Partial (only some with)	0/81 (0%)	1/18 (6%)	1/22 (5%)	0/16 (0%)	0/17 (0%)
Unsure	6/81 (7%)	0/18 (0%)	0/22 (0%)	0/16 (0%)	0/17 (0%)
Remarks	1 respondent did not answer			1 respondent did not answer	1 respondent did not answer 1 respondent had no CT scanner
MRI scanners with more than idle mode such as lower power modes					
Yes	45/81 (56%)	9/16 (56%)	11/20 (55%)	8/17 (47%)	8/18 (44%)
No	30/81 (37%)	6/16(38%)	8/20 (40%)	8/17 (47%)	9/18 (50%)
Partial (only some with)	0/81 (0%)	1/16 (6%)	1/20 (5%)	0/17 (0%)	0/18 (0%)
Unsure	6/81 (7%)	0/16 (0%)	0/20 (0%)	1/17 (6%)	1/18 (6%)
Remarks	1 respondent did not answer	2 institutions had no MRI scanner	2 institutions had no MRI scanner		1 institution had no MRI scanner
Paperless radiology requests and reports					
Yes	80/81 (99%)	10/18 (56%)	10/22 (45%)	10/17 (59%)	10/19 (53%)
No	1/81(1%)	8/18 (44%)	12/22 (55%)	7/17 (41%)	9/19 (47%)
Remarks	1 respondent did not answer	2 respondents which had partial (either requests or reports were paperless) were included in the ‘No’ group	3 respondents which had partial (either requests or reports were paperless) were included in the ‘NO’ group	1 respondent which had partial (either requests or reports were paperless) were included in the ‘NO’ group	2 respondents which had partial (either requests or reports were paperless) were included in the ‘NO’ group
Energy-conserving lighting: Motion-sensitive light and light-emitting diodes (LED) bulbs					
Yes	65/82 (79%)	9/18 (50%)	10/22 (45%)	5/16 (31%)	6/18 (33%)
No	17/82 (21%)	8/18 (44%)	11/22 (50%)	11/16 (69%)	12/18 (67%)
Partial (carpark, scan suites)	0/82 (0%)	1/18 (6%)	1/22 (5%)	0/16 (0%)	0/18 (0%)
Remarks				1 respondent did not answer	1 respondent did not answer
A recycling policy for non-contaminated waste (e.g. plastic bottles) in the workplace					
Yes	No separate answer	14/18 (83%)	18/22 (82%)	10/17 (59%)	10/19 (53%)
No	No separate answer	4/18 (22%)	4/22 (18%)	7/17 (41%)	9/19 (47%)
Remarks	Translated term incorporated recycling into the question for consideration for environmental factors in equipment procurement and/or service contract (proper waste management) decision-making process				

For other HIC training institutions, answers to environmentally conscious practices were above 50% except for CT scanners with auto-shutdown function (28%), for energy-conserving lighting (50%) and as high as 83% for a recycling policy for non-contaminated waste. For MIC training institutions, the figures were above 50% except for CT scanners with auto-shutdown function (31%), MRI scanners with more than idle mode (47%) and energy-conserving lighting (31%) with a high of 88% for regularly turning off workstations when not in use. This general trend was also noted when there was no differentiation from non-training institutions (Table 3).

#### Audit and research

In the training institutions, 18–23% conducted at least annual resource or energy conservation audits. Fifty-nine percent to 96% of these institutions did not have any officer or taskforce dedicated to sustainable radiology. Eighty-eight percent to 95% of these institutions did not conduct any research activities related to green radiology (Table 4).

**Table 4:** Audit and research: Japanese, other HIC and MIC institutions

Institution	Japanese training	Other HIC training	All Other HIC	MIC training	All MIC
Number	82	18	22	17	19
Are resource or energy conservation audits conducted at least annually?					
Yes	19/81 (23%)	2/18 (11%)	3/22 (14%)	3/17 (18%)	3/19 (16%)
No	61/81 (75%)	15/18 (83%)	18/22 (82%)	14/17 (82%)	16/19 (84%)
Unsure	1/81 (1%)	1/18 (6%)	1 (5%)	0/17 (0%)	0/19 (0%)
Remarks	One respondent did not answer this question				
Officer or taskforce dedicated to sustainable radiology					
Yes	2/82 (3%)	1/18 (6%)	5/22 (23%)	7/17 (41%)	7/19 (37%)
No	79/82 (96%)	17/18 (94%)	17/22 (77%)	10/17 (59%)	12/19 (63%)
Unsure	1/82 (1%)	0/18 (0%)	0/22 (0%)	0/17 (0%)	0/19 (0%)
Research activities in prior 12 months (Yes = 1 to 2 projects)					
Yes	4/82 (5%)	1/18 (6%)	1/22 (5%)	2/17 (12%)	2/19 (11%)
No	78/82 (95%)	17/18 (94%)	21/22 (95%)	15/17 (88%)	17/19 (89%)

### Societies: educational and academic activities under a radiological society or college

#### Demographics

In total, 15 radiology and nuclear medicine societies/colleges responded of which 9 respondents were only for their societies and 2 of these were affiliate societies to AOSR society members. Another 6 responses were on behalf of both their societies as well as the respective institutions they worked in. The institution responses have been included in the previous section under results for institutions. Eight societies from high-income countries/regions represented Australia/New Zealand, Hong Kong SAR, Japan, Macao SAR, Oman, Singapore, South Korea and Taiwan. Seven societies were from middle-income countries/regions—Indonesia, Malaysia, Myanmar, Thailand, Uzbekistan and Vietnam (World Bank GNI Per Capita, 2023). There were 2 societies from Thailand, one of which was an affiliate society. The response rate of only the AOSR society members was 54% (13/24).

#### Society-led efforts in green radiology

Thirty-three percent of societies had a working group, taskforce or committee that led and formulated sustainable radiology practice; 40% had academic activities consisting of 1 to 2 meetings in the past year and 27% reported sustainable radiology was a component of the radiology training curriculum (Table 5).

**Table 5:** Society-led green radiology efforts

Societies classification	HIC*	MIC**	All
Number	8	7	15
Working Group/Taskforce/Committee for sustainable radiology practice			
Yes	2/8 (25%)	3/7 (43%)	5/15 (33%)
No	6/8 (75%)	4/7 (57%)	10/15 (67%)
Number of academic activities pertaining to green and sustainable radiology over the past 12 months			
None	5/8 (63%)	4/7 (57%)	9/15 (60%)
1 to 2 meetings	3/8 (38%)	3/7 (43%)	6/15 (40%)
Sustainable radiology is a component of the radiology training curriculum			
Yes	3/8 (38%)	1/7 (14%)	4/15 (27%)
No	5/8 (63%)	6/7 (86%)	11/15 (73%)

*HIC high-income country/region

**MIC middle-income country/region

In the organization of conferences and courses, 53% of the societies selected the option to ‘minimise the use of paper’; followed by 40% for an online component; 33% each for hiring an environmentally conscious professional conference organizer and eliminating plastic disposable items (Table 6).

**Table 6:** Environmental impact factors considered important when organizing conferences and courses

Description	Number of times selected		
	HIC Societies (8)	MIC Societies (7)	All Societies (15)
Hiring a Professional Conference Organiser that considers environmental impact	2 (25%)	3 (43%)	5 (33%)
Minimise use of paper including advertising, invitations and programme book	4 (50%)	4 (57%)	8 (53%)
Eliminate bottled water and plastic disposable items such as utensils	2 (25%)	3 (43%)	5 (33%)
Reusing banners and backdrops	0 (0%)	1 (14%)	1 (7%)
Include on-line component to reduce travelling thereby reduce carbon footprint	4 (50%)	2 (29%)	6 (40%)

An additional comment for reducing the environmental impact for conferences/courses was to hold affiliated research meetings concurrently and collectively during main clinical conferences instead of separately at multiple venues and times. Another comment that was not specifically related to conferences, was to use Artificial Intelligence (AI) for simultaneous translation to reduce the need to have translators travel even during delivery and installation of equipment.

## Discussion

Our survey of current operations towards greener and more sustainable radiological practices across Asia-Oceania is the first of its kind in terms of receiving information from cross-regional institutions. As AOSR is comprised of society memberships with negligible number of individual members, we rely on the secretariat of each society member to distribute our survey to their respective constituents via variable communication channels (emails, social applications, and others), making it impossible to know the total number of surveys sent in most instances. The diversity of AOSR is such that membership in the main/national society is not necessarily compulsory within their region/country and neither is the purview of radiology training under the societies, instead these may be the governmental bodies such as ministries of health or higher education.

For this survey, we received twice as many responses from Japanese institutions compared to the rest of the Asia-Oceania. The availability of the survey in Japanese and the fact that one of the members of the SWG is a well-respected academic leader in Japan might have contributed to the robust responses from Japan. For these reasons, we decided to group the data from Japan separate from other regions/countries. Even though statistical analysis could not be performed without knowing the denominators for this survey, the information gleaned from this survey provides us better understanding of the current status and allows us to plan the way forward.

### Institution-led efforts in green radiology

Institution-only responses saw a diverse spread, from Oman to Tonga with a median of 1.5 institutions per country for countries other than Japan. As expected, all training institutions provided both in- and out-patient radiological services. For non-training institutions, half of them provided only out-patient radiological services.

#### Profile of high energy consumption equipment utilization

Our survey showed Japanese institutions overall had a high number (more than 3 units) of CT (91%), MRI (96%) scanners and IR suites (81%) whilst for the other HIC institutions even when only training institutions were considered, these figures ranged from 56 to 73% depending on the modality. Our data that Japan had a relatively high number of CT and MRI scanners is not unexpected as Japan has amongst the highest number of such scanners per million population (pmp) in the world (MRI 59.81pmp, CT 111.81pmp [11, 12]).

As expected, institutions with fewer scanners operated more days a week than institutions with more scanners. However, for IR suites the utilization pattern is less clear cut. The availability of radiologists with a high level of technical expertise required to perform interventional procedures may be a limiting factor for IR suite utilization and possibly account for this observation.

#### Energy and resource consumption

In Japan, only 54% of the institutions considered environmental factors for procurement and service contract decisions (such as for proper waste management). The Japanese survey had included recycling in the translated term for proper waste management, and therefore did not have a separate question and answer for recycling policies.

Japan ranked 7th at the top of all the Asia-Oceanian countries/regions in the International Energy Efficiency Scorecard 2022 [13]. In this survey, we see Japan’s strong energy efficiency policies reflected as the majority of their institutions used energy-conserving lights (79%) and turned off idle workstations (73%). Japan was also nearly completely paperless in radiology requests and reports, likely reflecting a more advanced healthcare information system. It was noted that CT scanners with auto-shutdown modes appeared most prevalent in Japan. These commendable survey findings underscore the importance of mature and technologically advanced health systems adopting green practices to balance against a relatively high density of CT and MRI scanners pmp.

For the other HIC and MIC institutions (whether training institutions were considered separately or otherwise), the majority of CT scanners were not optimised for energy-conservation. More can be done to ensure all future scanners have this feature or current scanners be upgraded where possible to auto-shutdown modes as the energy savings are considerable [14–16].

For lower power than idle modes in MRI scanners, generally institutions hover around the 50% threshold. Upgrading to lower power modes should be opted for, instead of just replacing with more eco-friendly ‘modern’ scanners. Reduction of non-productive (when not in use to scan patients) energy consumption reduces GHG emissions and costs of operation as well [6, 14, 15, 17]. Upgrading or being fitted with eco-friendly functions is better for the environment and contributes to the circular economy [7, 8], reducing the problem of waste disposal or the high ecological footprint of replacing with a new scanner.

MIC institutions had a relatively low adoption of energy conserving lights (about a third) and this could be attributed to the higher cost of acquisition of these lights, where the budget may be prioritized for equipment purchase and maintenance. Yet, a positive feature is that 88–89% of only training and all MIC institutions turned off idle workstations, as this simple action also saves considerable energy [14].

A high majority (82%) of other HIC institutions had a recycling policy for non-contaminated waste in the workplace. The MIC institutions had a smaller majority (59% of MIC training and 53% all MIC institutions). All institutions should be encouraged to separate their waste and have recycling policies [18, 19].

#### Audit and research

An overwhelming majority of respondents from all institutions including Japan, indicated that there were neither regular annual audits of energy conservation, organised workgroups or officers overseeing sustainability, nor sustainability related research activities. We therefore recommend an urgent and deliberate push among institutional radiology leadership to formulate strategies for driving sustainability in their own practices [7, 8]. For example, strategies to reduce inappropriate imaging cuts unnecessary imaging, thereby reducing the carbon footprint, the environmental impact of contrast media and overall costs of healthcare [18–20].

### Society-led efforts in green radiology

Based on our survey, only 33% of the societies have set up a working group (committee or taskforce) that leads and formulates practices for sustainable radiology. Only 40% had conducted 1 to 2 academic activities pertaining to sustainability in the past 12 months. Four societies reported that sustainable radiology is already a component of their training curriculum. These findings are not surprising, given the relatively nascent nature of sustainability as an area of concern in radiology. That said, not all societies/colleges have oversight of the training curriculum.

Annual scientific meetings are highly visible opportunities for promoting sustainability, not just as a theme in a scientific track but also in meeting logistics, such as transitioning from paper to digital conference materials. In our survey, minimising use of paper was considered most important, followed by the provision of online participation to reduce physical travel. The Covid-19 pandemic has uncovered the benefits of online participation, reducing travel-related GHG emissions and financial costs. Other measures include hiring an environment-conscious professional conference organizer and eliminating plastic waste, though these were ranked lower in priority by our respondents. Stage backdrops where possible, should be electronic and reusable.

A comment worth noting, although not specific for conferences, is that generative AI translation could overcome language barriers and obviate physical attendance by human translators to attend training sessions such as those held by vendor application specialists during delivery and equipment installation. This would be a relatively common application of AI as a support tool that would be very helpful to reduce GHG emissions from travel. This is distinct from higher computing requirements introduced by widespread integration of AI into radiological practices including the conduct of conferences/courses, which could paradoxically lead to higher energy use and an even larger carbon footprint [21].

## Limitations

Given the large size and diversity of Asia-Oceania, a time-limited survey such as ours is unlikely to fully represent the practice patterns of all healthcare systems among our member societies and colleges. Apart from the caveats mentioned in the results section, other limitations are listed below.

1. There was a lack of data from China and India, the two most populous countries in Asia-Oceania.
2. The language and survey bias was not fully anticipated. Whilst translation to the primary language of a country/region is usually likely to encourage more responses, the additional active support of a society member in the distribution of the survey cannot be underestimated. This resulted in an excellent response from Japanese institutions, thereby skewing the data markedly.
3. Response bias, as this is a self-reported survey. Respondents may be tempted to give an answer which reflects better on their institution or society. However, the anonymized presentation of the data with no individual level information may have helped to mitigate this tendency.
4. A proper statistical analysis was not possible in view of the heterogeneity of data, the nature of the survey and its distribution method. Training institutions typically differ from non-training institutions in terms of scale, workflow and level of throughput. To mitigate this, in assessing the environmental impact of radiology service providers, comparison was limited and differentiation was made between training and non-training institutions.
5. As for the utilization of scanners, there may be overestimation of the utilization if there are multiple scanners in the institution, such as when 1 out of 6 scanners operate throughout the night.
6. Inherent issues with translations. The nuances of translation and that sometimes, direct translation is not possible, led to initial assumption that the Japanese survey did not have the question related to recycling when viewed after translation back to English. Even so, direct comparison is therefore no longer possible as the question had a slightly modified and expanded meaning for proper waste management in the question ‘environmental considerations in equipment procurement and service contract decisions’ once translated to Japanese.
7. The survey findings are more likely to reflect the practice of sustainability in radiology in public healthcare, which tend to be less heterogeneous than private radiology services in most countries. Yet, sustainability is an agenda for all, and urgent action by private radiology providers to prioritise efforts is equally important.
8. Due to the broad scope of environmental sustainability as a topic, our survey was not able to cover many aspects that are worth deeper consideration. The pollution of surface water by contrast media attributed to the inappropriate use of contrast enhanced CT and MRI is one such example. We had to balance between being comprehensive and administering a survey that was brief enough to elicit a response rate that reflects current practices.
9. Lastly, we are aware that sustainability does not just refer to the environment, be it carbon footprint or waste. It also encompasses ideals such as health equity (social) and leadership (governance). We chose to focus on environmental sustainability as a low-hanging fruit for AOSR to rally member societies/colleges to act upon.

## Conclusion

Despite the limitations, the survey presents AOSR with the opportunity to identify role model countries/regions that can assist to spread best practices in energy conservation in their different ways. As the awareness and impetus to implement an environmentally sustainable practice seemed mixed, much can be done to empower all stakeholders, be it in the provision of services or in training. The lower resourced areas can benefit immensely from our collective efforts and ‘role models’ to promote environmental friendliness, even as they strive to rapidly increase access to diagnostic imaging and image guided interventions.

AOSR is committed to facilitating the sharing of best practices in green radiology. We believe that we can contribute by influencing our radiology leaders to urgently formulate strategies and implement policies that reduce our environmental impact. We would like to encourage academic efforts in this regard. As a professional society, we have the mandate to partner with our counterpart societies and industry stakeholders, to emphasise the importance of sustainability as a critical agenda, through our various activities. An actionable checklist that was formulated partly based on the findings of the survey has been included as supplement B.

## Supplementary Information

Below is the link to the electronic supplementary material.

Supplementary Material A

Supplementary Material B
