# Supplementary material for: The Asian Oceanian Society of Radiology (AOSR) green radiology survey: a catalyst for action
Source: Jpn J Radiol. 2025 Dec 23;44(4):607–17. doi: 10.1007/s11604-025-01918-y (PMC13038643; doi:10.1007/s11604-025-01918-y)
Supplement: Supplementary file 1 — Supplementary Material A [file 11604_2025_1918_MOESM1_ESM.pdf]

AOSR
The Asian Oceanian Society of Radiology

The Asian Oceanian Society of Radiology (AOSR) Green Radiology Survey: A Catalyst for Action

Supplement A: The AOSR Green Survey Questionnaire

Introduction

Increased emission of greenhouse gases resulting in the phenomenon of global warming is not news to any of us. About 4.4% of global net CO2 emissions come from the healthcare sector, yet medicine as a whole is slow to adopt sustainable practices.

Within radiology, we are well accustomed to the "as low as reasonably achievable" principle, or ALARA, referring to the radiation exposure to a patient undergoing a particular study or procedure should be as low as reasonably possible. Have you considered taking a similar approach to environmental impact?

Green radiology is a sustainable, innovative, and responsible approach in radiology practice that focuses on minimising the negative environmental impact of our technologies and procedures. The primary goal is to reduce the carbon, water and ecological footprint.[1]

Through this survey, AOSR hopes to gain a deeper understanding of your operation so that we can make meaningful steps toward achieving greener and more sustainable radiological practices. By voluntarily participating in this short survey, you grant permission for the data generated from this survey to be used for possible project or publication.

1. Lojo-Lendoiro S, Rovira A, Morales Santos A. Green radiology: How to develop sustainable radiology. Radiología (English Edition) 66(3) 2024: 248-259.
2. Chaban YV, Vosshenrich J, McKee H, et al. Environmental Sustainability and MRI: Challenges, Opportunities, and a Call for Action. JMRI 2024; 59:1149-1167.
3. Schoen JH, Burdette JH, West TG, et al. Savings in CT Net Scan Energy Consumption: Assessment Using Dose Report Metrics and Comparison With Savings in Idle State Energy Consumption. AJR 2024; 222:e2330189.

- **If you are representing your institution, please answer questions 1 – 4**
- **If you are representing a College/Society, please answer question 1 and 5**
- **If you are able to represent both your institution and your College/Radiological Society, please answer all questions**

1. About you:

a. Please provide the name of your institution (imaging center or hospital) and country and/or the College/Radiological Society you represent.
<Free text>

b. Please provide your position in the radiology department or the College/Radiological Society you represent.
<Free text>

2. About your institution:

a. My institution provides radiological service for:
- ☐ Out-patient only
- ☐ In-patient only
- ☐ In-patient and out-patient

b. How many MRI scanners does your institution have?
- ☐ 0, None
- ☐ 1-2
- ☐ 3-4
- ☐ 5-6
- ☐ >6

c. On average, each MRI operates how many days per week?
- ☐ 0, No MRI scanner
- ☐ ≤2
- ☐ 3-5
- ☐ 6-7

d. On average, each MRI operates how many hours per operating day (for routine bookings)?
- ☐ 0, No MRI scanner
- ☐ ≤8
- ☐ 9-16
- ☐ >16

e. How many CT scanners does your institution have?
- ☐ 0, None
- ☐ 1-2
- ☐ 3-4
- ☐ 5-6
- ☐ >6

f. On average, each CT operates how many days per week?
- ☐ 0, No CT scanner
- ☐ ≤2
- ☐ 3-5
- ☐ 6-7

g. On average, each CT operates how many hours per operating day (for routine bookings)?
- ☐ 0, No CT scanner
- ☐ ≤8
- ☐ 9-16
- ☐ >16

h. How many Interventional Radiology suites (including operating theatres with angiography) does your institution have?
- ☐ 0, None
- ☐ 1-2
- ☐ 3-4
- ☐ 5-6
- ☐ >6

i. On average, each Interventional Radiology suite operates how many days per week?
- ☐ 0, None
- ☐ ≤2
- ☐ 3-5
- ☐ 6-7

j. On average, each Interventional Radiology suite operates how many hours per operating day (for routine bookings)?
- ☐ 0, None
- ☐ ≤8
- ☐ 9-16
- ☐ >16

3. Energy and resource consumption:

a. Are environmental requirements considered during acquisition (such as energy efficiency) and/or service contract (proper waste management) decision-making process?
- ☐ Yes
- ☐ No
- ☐ Other: <free text>

b. Are your workstations regularly turned off when not in use?
- ☐ Yes
- ☐ No
- ☐ Other: <free text>

c. Are the CT scanners incorporated with auto-shutdown functions?
- ☐ Yes
- ☐ No
- ☐ Other: <free text>

d. Are the MRI scanners incorporated with more than idle mode (automatic state between active scanning) – such as low power modes (standby, sleep mode)?
- ☐ Yes
- ☐ No
- ☐ Other: <free text>

e. Are the radiology request and reporting paperless?
- ☐ Yes
- ☐ No
- ☐ Other: <free text>

f. Are there motion-sensitive light and light-emitting diodes (LED) bulbs in the workplace?
- ☐ Yes
- ☐ No
- ☐ Other: <free text>

g. Is there a policy / practice of recycling non-contaminated waste (e.g. plastic bottles) in the workplace?
- ☐ Yes
- ☐ No
- ☐ Other: <free text>

4. Auditing & Research

a. Are resource and/or energy conservation audits performed?
- ☐ Yes, interval: <free text>
- ☐ No

b. Is there a subject officer or taskforce dedicated to sustainable radiology practices?
- ☐ Yes
- ☐ No
- ☐ Other: <free text>

c. Over the past 12 months, how many research activities pertained to the topic of green and sustainable radiology?
- ☐ 0
- ☐ 1-2 project(s)
- ☐ ≥3 projects

5. Education & Academic activities under College/Radiological Society

a. Is there a working group/taskforce/committee that leads and formulates the practice of sustainable radiology?
- ☐ Yes
- ☐ No
- ☐ Other: <free text>

b. Over the past 12 months, how many academic activities pertained to the topic of green and sustainable clinical radiology?
- ☐ 0
- ☐ 1-2 meetings
- ☐ ≥3 meetings

c. Is sustainable radiology practices a component of radiology training curriculum?
- ☐ Yes
- ☐ No
- ☐ Other: <free text>

d. When organising academic activities including conferences and courses, the below factors are considered important:
- ☐ Hiring a Professional Conference Organiser that considers environmental impact
- ☐ Minimise use of paper including advertising, invitations and programme book
- ☐ Eliminate bottled water and plastic disposable items such as utensils
- ☐ Reusing banners and backdrops
- ☐ Include on-line component to reduce travelling thereby reduce carbon footprint
- ☐ Other: <free text>

This ends the survey. Thank you so much!

The Asian Oceanian Society of Radiology
71 Yangjaecheon-ro, Seocho-gu, Seoul, 06754 Korea
Tel: (82)-31-704-4261 / office@aosr.kr
