# Supplementary material for: The Asian Oceanian Society of Radiology (AOSR) green radiology survey: a catalyst for action
Source: Jpn J Radiol. 2025 Dec 23;44(4):607–17. doi: 10.1007/s11604-025-01918-y (PMC13038643; doi:10.1007/s11604-025-01918-y)
Supplement: Supplementary file 2 — Supplementary Material B [file 11604_2025_1918_MOESM2_ESM.pdf]

The Asian Oceanian Society of Radiology (AOSR) Green Radiology Survey:
A Catalyst for Action
Supplement B

AOSR Sustainability in Radiology Actionable Checklist for Healthcare Institutions and Radiological Societies
Formulated partly based upon the AOSR Green Radiology Survey Responses

I. Healthcare Institutions (Radiology Departments & Hospitals)

1. Governance & Strategic Planning

[ ] Establish formal sustainability policies at departmental and hospital levels
[ ] Designate sustainability leads or committees within radiology units
[ ] Integrate sustainability metrics into departmental KPIs and performance reviews
[ ] Align procurement and capital planning with environmental goals

2. Operational Optimization

[ ] Implement waste reduction protocols, including proper disposal of radiological waste
[ ] Transition to paperless workflows (e.g., digital logbooks, e-referrals, PACS optimization)
[ ] Conduct regular stock audits to minimize over-ordering and expired inventory
[ ] Optimize imaging protocols to reduce unnecessary scans and repeat procedures

3. Equipment & Infrastructure

[ ] Prioritize acquisition of low-helium and energy-efficient imaging equipment
[ ] Evaluate lifecycle energy consumption of AI platforms and digital systems
[ ] Maximize use of existing equipment while planning phased upgrades to eco-friendly models
[ ] Explore intelligent energy management solutions (e.g., smart HVAC, auto-shutdown systems)

4. Education & Staff Engagement

[ ] Develop training modules on sustainable radiology practices
[ ] Conduct internal awareness campaigns on environmental impact and best practices
[ ] Foster cross-disciplinary collaboration on sustainability (e.g., with infection control, IT)

5. Data Collection & Research

[ ] Establish baseline metrics on energy use, waste generation, and imaging volumes
[ ] Initiate or participate in research on sustainability interventions and outcomes
[ ] Share findings with national bodies and radiological societies to inform broader policy

II. Radiological Societies

1. Leadership & Advocacy

[ ] Promote sustainability as a strategic priority in society communications
[ ] Collaborate with ministries of health to develop national SOPs for sustainable radiology
[ ] Advocate for funding and infrastructure support for eco-friendly upgrades

2. Education & Community Engagement

[ ] Integrate sustainability topics into CME programs and certification pathways
[ ] Organize workshops and webinars on sustainable imaging, procurement, and governance
[ ] Encourage adoption of electronic logbooks and digital training platforms

3. Congress & Event Sustainability

[ ] Enforce green congress policies (e.g., no plastic bags, reusable materials, digital-only handouts)
[ ] Partner with vendors to design eco-conscious booths and promotional materials
[ ] Recognize and reward sustainability initiatives by member societies

4. Monitoring & Collaboration

[ ] Conduct periodic surveys to assess sustainability adoption and challenges
[ ] Facilitate regional knowledge exchange on best practices and innovations
[ ] Publish case studies and toolkits to support implementation across income levels

III. Cross-Cutting Recommendations

[ ] Develop a regional sustainability framework tailored to income-level diversity
[ ] Create a repository of validated tools and guidelines for sustainable radiology
[ ] Explore partnerships with environmental NGOs, academic institutions, and tech innovators
[ ] Address medicolegal and infection control concerns through evidence-based guidance
[ ] Promote research on the carbon footprint of digitalization and AI in radiology
